# Sex Differences in Electronic Health Record Navigation Strategies: Secondary Data Analysis

**DOI:** 10.2196/25957

**Published:** 2021-06-24

**Authors:** Daniel R Seifer, Karess Mcgrath, Gretchen Scholl, Vishnu Mohan, Jeffrey A Gold

**Affiliations:** 1 Division of Pulmonary and Critical Care Medicine Oregon Health & Science University Portland, OR United States; 2 Department of Medical Informatics and Clinical Epidemiology Oregon Health & Science University Portland, OR United States

**Keywords:** eye tracking, electronic health record, gaze, usability, sex differences

## Abstract

**Background:**

Use of electronic health records (EHRs) has increased dramatically over the past decade. Their widespread adoption has been plagued with numerous complaints about usability, with subsequent impacts on patient safety and provider well-being. Data in other fields suggest biological sex impacts basic patterns of navigation in electronic media.

**Objective:**

This study aimed to determine whether biological sex impacted physicians’ navigational strategies while using EHRs.

**Methods:**

This is a secondary analysis of a prior study where physicians were given verbal and written signout, and then, while being monitored with an eye tracker, were asked to review a simulated record in our institution’s EHR system, which contained 14 patient safety items. Afterward, the number of safety items recognized was recorded.

**Results:**

A total of 93 physicians (female: n=46, male: n=47) participated in the study. Two gaze patterns were identified: one characterized more so by saccadic (“scanning”) eye movements and the other characterized more so by longer fixations (“staring”). Female physicians were more likely to use the scanning pattern; they had a shorter mean fixation duration *(P=*.005), traveled more distance per minute of screen time (*P*=.03), had more saccades per minute of screen time (*P*=.02), and had longer periods of saccadic movement (*P*=.03). The average proportion of time spent staring compared to scanning (the Gaze Index [GI]) across all participants was approximately 3:1. Females were more likely than males to have a GI value <3.0 (*P*=.003). At the extremes, males were more likely to have a GI value >5, while females were more likely to have a GI value <1. Differences in navigational strategy had no impact on task performance.

**Conclusions:**

Females and males demonstrate fundamentally different navigational strategies while navigating the EHR. This has potentially significant impacts for usability testing in EHR training and design. Further studies are needed to determine if the detected differences in gaze patterns produce meaningful differences in cognitive load while using EHRs.

## Introduction

In the last two decades, the percentage of physicians using an electronic health record (EHR) in their practice has risen from around 10% to over 80%, driven in varying degrees by federal initiatives, intra- and internetwork pressure, and perceived potential advantages. This increase represents a broad shift in practice patterns that have introduced new challenges into the process of delivering quality medical care. Though EHRs come with many benefits such as improved compliance with clinical guidelines, substantial decreases in medication errors, the ability to improve care through simulation, and increased cost-savings at an institutional level, the development and implementation of the EHR has far outpaced investigation into their downsides [1-5]. It is clear that the amount of documentation required per patient has increased substantially with the advent and implementation of EHR systems [6]. Interface design, usability, and interoperability issues have plagued EHRs since their initial iteration in the 1970s; increased documentation and input requirements correlate with increased error rates, longer hospital stays, less job satisfaction, and physician burnout [7-10]. Tools for understanding the usability patterns of EHRs are sorely needed in order to better inform system design and provider training.

The user experience of any type of electronic system such as an EHR depends entirely around the ways in which the user navigates the system to produce desired outcomes; an eminently capable program will be useless if users cannot utilize its capabilities. Appropriately then, interface navigability is a consideration at the forefront of electronic system design. Of course, humans did not evolve with electronic system navigation as an active evolutionary pressure; instead, the human brain is optimized for spatial navigation. There are two recognized spatial navigation strategies: (1) local landmark usage and (2) distant cues (eg, cardinal directions) utilization [11]. All neurotypical humans are capable of utilizing either strategy when required by a given situation; however, individuals have a reliable tendency to default to one strategy over the other, and sex is a strong predictor of one’s default strategy [11-15]. In these spatial tasks, females tend to default to using local landmarks while males default to utilizing distal cues. Although social expectations can have an effect on task performance, the phenomenon of default strategy selection appears to be strongly influenced by genetic and hormonal factors rather than socialization [16-19].

Analogous sex differences in navigational strategy have also been observed in the varied contexts of navigating electronic screens. Females reported higher utilization of situation-specific navigational cues during single-site hypertextual (ie, website) navigation and more frequently used landmarks to guide others verbally when acting as instructors [20]. Previous work showed that performance on text-based database querying tasks (eg, Google, PubMed, etc) improves with increased content knowledge across both sexes; however, no matter the level of content knowledge, elementary-school–aged females spent more time reading documents, formulated fewer overall queries, and clicked on fewer links per minute than their male counterparts, although none of these measures correlated with overall task performance [21]. Constrained to a search task in an area of low-domain knowledge, middle-school–aged males and females showed no differences in performance in nonelectronic library searches but did show a difference in task performance when using the web, a finding which has been replicated [22,23]. Finally, differences in gaze patterns during the higher-level analytical tasks of C-program debugging correlated with task performance [24,25].

Only a handful of studies have investigated eye tracking with regard to EHR usage with the majority focused on EHR use as opposed to characterizing user characteristics [26-28]. Previously, our group used eye tracking to characterize general features of gaze in hospital physicians and found that there were two distinct workflows: while all participating physicians spent far more time writing their notes than perusing the chart overall, one group of physicians perused the chart at length before opening their notes while the other started their notes immediately. This difference in workflow was associated with physician sex in that females physicians were more likely to peruse the chart before starting their notes [29]. Though no sex-related performance differences in medical diagnostic reasoning have been reported thus far, these results showed that differences in EHR navigation do exist between female and male physicians. While the impact of sex differences on EHR navigation remains unknown, what is apparent is that the increase in EHR use nationally has correlated with a dramatic increase in physician burnout that is increasingly being recognized as disproportionately affecting female physicians [9,10,30].

Simulation affords a powerful tool to better understand the role of sex in EHR navigation by removing the variability inherent in analyzing deidentified patient charts and replacing it with a standardized, validated case that is the same for all participants. We have previously reported the use of high-fidelity EHR-based simulation to understand error recognition in simulated intensive care unit (ICU) charts [28,31-33]. In these preliminary studies, we incorporated eye and screen tracking to determine whether gaze metrics could be used as surrogates for EHR performance. We demonstrated that a number of eye-tracking metrics correlated with the recognition of embedded safety items within the chart for the entire cohort. The goals of this study were twofold: (1) to expand upon the initial data set in order to analyze whether sex was associated with differential navigation patterns during simulated EHR exercises, and (2) to determine the impact, if any, this difference had on task performance.

## Methods

This study was approved by the Institutional Review Board at Oregon Health & Science University. The study was deemed minimal risk, and formal informed consent was not required. However, all participants were provided with an information sheet on our research protocol.

Physicians’ eye movements were recorded with a Tobii X1 monitor–mounted Light Eye Tracker (Tobii Systems) while completing a previously validated EHR-based simulation exercise in our EHR environment (EpicCare, Epic Systems Inc) [4,33]. This simulated instance imported all end-user settings, preferences, and customized screens so that participants were able to use their own personalized data gathering tools. We created 2 simulated ICU patients, each admitted to the ICU for 5 days. We attempted to make the simulated patient scenarios as robust as possible, with hourly vital signs, lab results, and nursing, as well as resident and attending notes. Residents and fellows rotating through the medical ICU were eligible for enrollment, and thus all had experience with the use of the EHR in the context of the exercise they were being asked to complete. Participants were provided a written “signout” and then given 10 minutes to review data on one of the simulation cases, with instructions to review the EHR as if they were assuming care for the patient and would be presenting them on rounds, along with any potential changes in management that would be required for their care. At the end of 10 minutes, participants then presented the care plan for the patient to a member of the study team. The participants’ performance was assessed by the number of safety issues they verbalized during this presentation, followed by a debriefing of the safety issues associated with the case as previously described [4,31,32]. The physical nature of the computer station used for the testing was standardized by fixing monitor, desk, and chair height, as well as tilt and relative positioning of the monitor at 65 cm from the participant. Calibration was performed for each subject using a 1-minute–long, 9-point calibration algorithm provided by the manufacturer.

Once complete, all videos were captured and analyzed with the vendor’s eye-tracking software (Tobii Studio, Tobii Systems). Fixations were defined as a period (>60 milliseconds) in which providers’ eyes tracked without eye velocity exceeding 30 degrees/second. Saccades (rapid scanning eye movements) were defined as periods with gaze velocity exceeding 30 degrees/second, these definitions being adapted from current standards within the literature [34]. Static eye tracking on points for <60 milliseconds were termed “microfixations,” as they did not meet broadly acceptable standards for the fixations referenced above. These were present primarily while participants were retrospectively observed to be reading blocks of full sentences. Raw tracking data was analyzed using custom Excel macros designed to calculate more complex variables (eg, screen distance between fixation points, fixation duration, etc). Variable distribution was analyzed in GraphPad (GraphPad Software Inc). Many of the recorded variables were definitionally bounded on the low end producing one-tailed variable distributions. Given these distributions, appropriate nonparametric tests were used to assess for differences between group means.

Krejtz et al [35] introduced coefficient Κ, seeking to construct a single, trackable indicator of fluctuation between ambient or focal eye movements. This metric was designed to track gaze patterns across visual stimuli (eg, art, visual stimulation tasks, etc), and their reported results are promising; however, it was not suitable for answering the question posed in our work, given that we sought to differentiate between two active self-driven data extraction strategies (“scanning” vs “staring”), which requires a variable built to purpose [35]. We thus developed a novel variable—the Gaze Index (GI)—to denote the ratio of time spent in long fixation (“staring”) to time spent in saccadic motion (“scanning”). The GI represents the proportion of time each participant spent in fixation versus saccade. For example, a GI of 0.11 would indicate that a participant spent approximately 9 times more of their screen time in saccade-microfixation-saccade-etc (a “reading”/“scanning” pattern) than in full fixation (“staring”). It inherently normalizes for the time each participant spent viewing the screen, making the GI a superior representation of participant behavior when compared to the raw ratio of fixations to saccades. Normalization to screen time is particularly important given the top-bounded nature of saccadic movement compared with the unbounded upper limit of fixation periods, which could easily skew results based on the finite nature of this time-constrained task.

For analysis, we correlated individual usability measures to performance on the simulation. Differences in performance between groups with specific usability characteristics were compared using a Mann-Whitney *U* Test based on the size of the data set and the nonparametric nature of the data. Normality was assessed via Kolmogorov-Smirnov. All analyses were performed using GraphPad Prism (GraphPad Software Inc), and a *P* value of <.05 was considered statistically significant.

## Results

The data set comprised the eye-tracking recordings of 93 physicians (female: n=46, male: n=47). Of these, 39 (42%) were interns; the remaining were in their PGY (postgraduate year) 2 year and above. None of the recordings were excluded from analysis. Self-supplied specialty identifications were similar between groups (Table 1).

**Table 1:** Demographics of study participants (N=93).

Specialty	Females (n=46), n (%)	Males (n=47), n (%)
Internal Medicine	34 (74)	32 (68)
Anesthesia	3 (7)	3 (6)
Pulmonary & Critical Care	3 (7)	2 (4)
Critical Care	3 (7)	3 (6)
Emergency Medicine	1 (2)	2 (4)
Neurology	1 (2)	0 (0)
Unspecified	1 (2)	5 (11)

**Figure 1:**
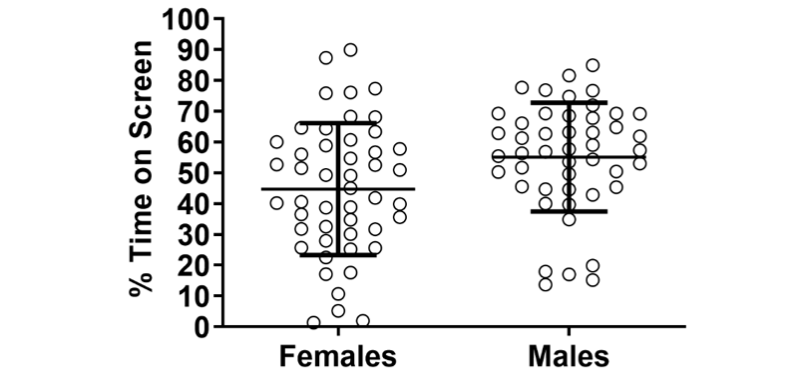
Comparison of percent task time spent looking at the screen between female and male physicians. Each subject is represented by an individual point with the error bars representing mean (SD).

Overall, females spent less task time looking at the screen than males (44.7% vs 55.1%; *P*=.007, *U*=767) (Figure 1).

**Figure 2:**
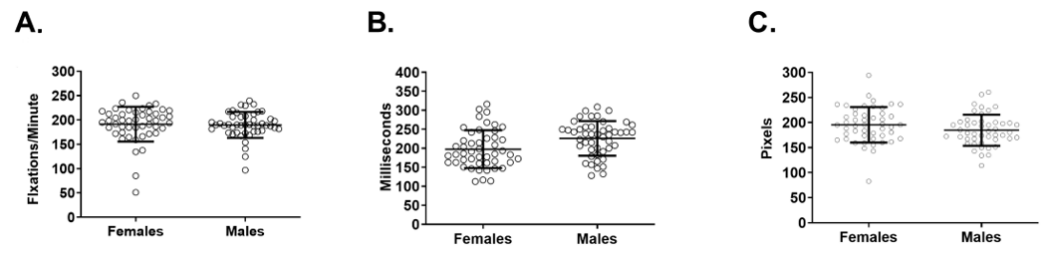
Comparison of fixation data metrics between female and male physicians. (A) There was no difference in the number of fixations recorded per minute of screen time. (B) Female physicians had a significantly lower mean fixation duration than their male colleagues. (C) There was a trend toward female physicians traveling a longer distance between fixation points. Error bars represent mean (SD).

While looking at the screen, the mean fixation (“stare”) duration of all participants was 212 milliseconds, which is in line with previously reported mean fixation durations during text-reading tasks [36]. No difference was found in the number of fixations per minute between females and males (mean 191.3, SD 5.1 vs mean 189.7, SD 3.9) (Figure 2A); however, the mean fixation duration was shorter for females than males (mean 197.6, SD 7.2 vs mean 226, SD 6.6 milliseconds; *P*=.005, *U*=740) (Figure 2B). Finally, there was a trend toward an increase in the mean distance traveled between fixations among females (mean 195.7, SD 5.1 pixels vs mean 184.6, SD 4.5 pixels; *P*=.07, *U*=892) (Figure 2C).

**Figure 3:**
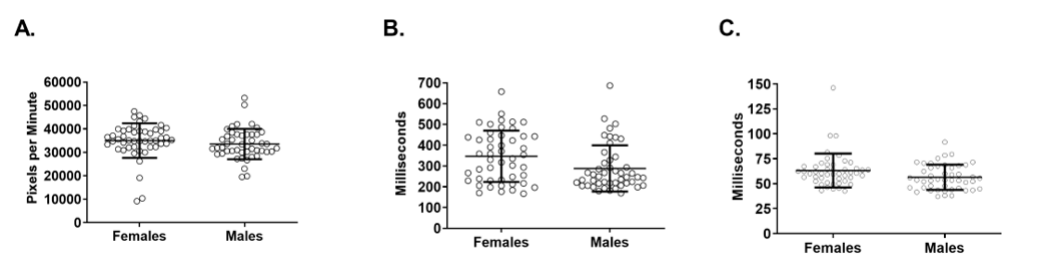
Comparison of saccade metrics between female and male physicians. (A) Female physicians traveled more screen distance per minute of saccadic movement than their male colleagues. (B) Per minute of screen time, female physicians had more, separate, and distinguishable periods of saccadic movement than their male colleagues. (C) The saccadic periods of female physicians were also significantly longer than those of their male peers. Error bars represent mean (SD).

We next analyzed saccadic (“scanning”) eye movements. Females traveled more distance per minute of screen time (35,026, SD 1065 pixels per minute vs 33,542, SD 943 pixels per minute; *P*=.03, *U*=839) (Figure 3A) and had more saccades per minute of screen time (347, SD 18 vs 288, SD 16; *P*=.02, *U*=817) (Figure 3B). Furthermore, their periods of saccadic movement were of longer duration (63.1, SD 2.4 milliseconds vs 56.4, SD 1.8 milliseconds; *P*=.03, *U*=836) (Figure 3C). In total, female physicians scanned more often and for longer durations than their male counterparts.

**Figure 4:**
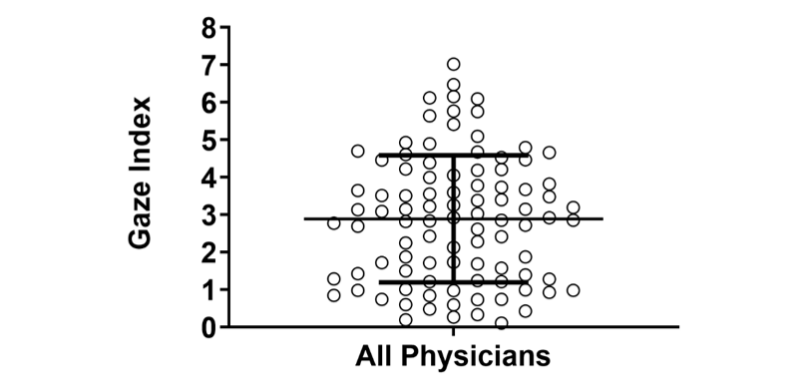
Plot of the Gaze Index (GI) of all participants. The mean GI of all participants was approximately a 3:1 ratio, indicating that the participants, on average, spent 3 times as much of their screen time staring as they did scanning: 46 participants had a GI value >3.0, while 47 participants had a GI value <3.0. Error bars represent mean (SD).

In order to investigate the relative time spent between fixation and saccadic movement, we calculated GI values for all subjects. The GI had a wide frequency distribution across all participants (0.11-7.02). The mean GI of all participants was approximately a 3:1 ratio, indicating that the participants, on average, spent 3 times as much of their screen time staring as they did scanning: 46 participants had a GI value >3.0, while 47 participants had a GI value <3.0 (Figure 4).

**Figure 5:**
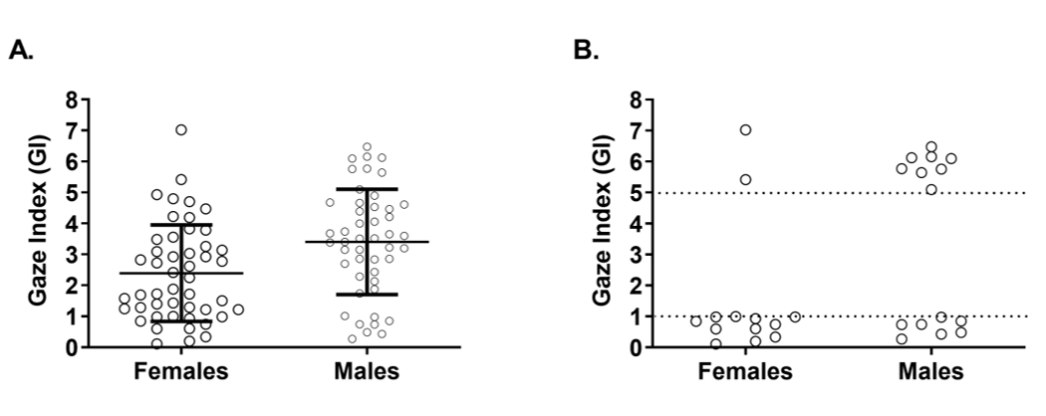
Comparison of the Gaze Index (GI) of female and male physicians. (A) The GI differed significantly between female and male physicians with the former having a group average of <3 and the latter having a group average of >3. (B) “Superstarers” (those with a GI>5, closed circles) were disproportionately male (odds ratio [OR] 0.22 vs OR 4.51), while “superscanners” (those with a GI<1, open circles) were disproportionately female (OR 1.58 vs OR 0.63). Error bars represent mean (SD).

We termed those users with a GI>3 “starers” and those with GI<3 “scanners” to qualify their tendency toward either eye movement relative to the average GI of 3. Interestingly, females were more likely than males to have a GI<3.0 (2.4 vs 3.4; *P*=.004, *U*=739) (Figure 5A). When looking at GI extremes, males were more likely to have a GI>5 (odds ratio [OR] 4.51 vs OR 0.22) while females were more likely to have a GI<1 (OR 1.58 vs OR 0.63) (Figure 5B). No differences between specialties were detected either in pooled analysis or in analysis within sex groups (not shown).

**Figure 6:**
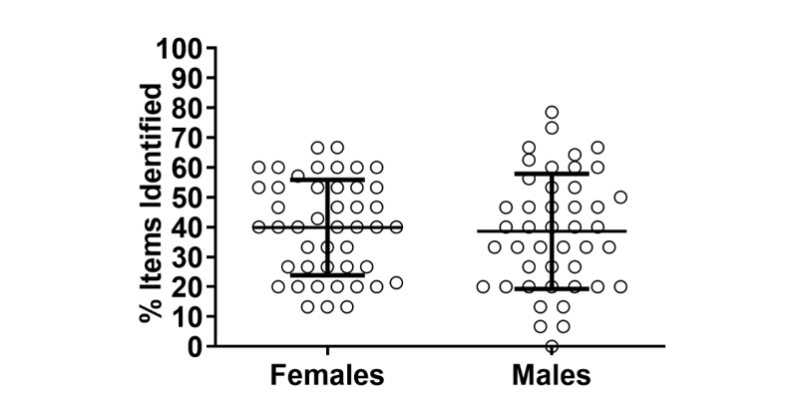
Comparison of task performance between female and male physicians; no difference was detected. Error bars represent mean (SD).

Lastly, we sought to determine whether differences in navigation patterns predicted performance in the simulation. Overall, there were no appreciable differences between females and males in task performance measured by percentage of existing safety items identified (39.9, SD 16% vs 38.6, SD 19%; *P*=.97) (Figure 6). Similarly, when users were grouped by their differences in GI rather than by sex (GI<1 vs GI>5; GI<3 vs GI>3) there were no detectable differences in task performance (not shown).

## Discussion

### Principal Findings

This study characterized the eye movements of physicians as they completed a previously validated simulation of prerounding data collection, and showed that physician sex was associated with distinctly different patterns of eye movements while reviewing the EHR. Female physicians tended to scan the medical chart for both proportionally longer amounts of time and more frequently than their male colleagues. By contrast, male physicians spent more of their EHR task time staring at points of interest for longer periods. No other variable was correlated with physician GI, and neither physician sex nor any gaze metric, including the GI, was predictive of task performance.

Scanning (saccades) and staring (fixations) are inextricably linked in gaze mechanics. One cannot scan indefinitely and still process information nor can one fixate indefinitely if they wish to move between points of interest. As such, a focus on either type of movement alone would not fully characterize the overall gaze pattern. For example, a high number of saccades per minute coupled with a high number of short fixations (as is seen while reading text) would be a completely different pattern than a high number of saccades per minute coupled with a smaller number of longer fixations (as is seen while perusing quantitative charts). Similarly, a simple ratio of the absolute number of fixations divided by the absolute number of saccades would be inadequate to describe a gaze pattern as the duration of both movement portions would not be incorporated into this ratio. For the purposes of correlative analysis then, a single variable taking into account both movement types and their respective durations is required. In this study, the GI served this purpose and allowed us to assess whether overall gaze pattern is associated with any other outcome such as task performance.

Our finding that sex is predictive of differences in physician GI during EHR usage is consistent with work in other navigatory domains—both spatial and electronic [11-15,20-22,25]. When viewed as a whole, the work done in this area supports that sex-linked navigatorial differences are global and have at least part of their roots in biology as opposed to being solely linked to socialization or environmental cues [17-19]. A GI of <3 is descriptive of a scanning pattern of EHR analysis, examples of which may include reading text in notes, sequentially scanning the entirety of a flowsheet, etc. This gaze pattern is even more strongly represented in those physicians with GI<1, a group predominantly female. In contrast, a GI value of >3 describes a fixation-focused pattern of EHR analysis inconsistent with text reading and more consistent with a longer examination of fixed data points without reading the sheet in entirety; this is even more true of those with a GI>5, who were overwhelmingly male. Our finding that female physicians’ eye movements represented greater screen distance per time than their male counterparts while they maintained the same absolute number of fixations per time also supports this characterization of female gaze patterns as consistent with linear, sequential scanning.

Our findings thus align with the differences between the sexes observed in the spatial realm where females default to scanning local landmarks for orientation while males default to fixating on distant orienting points. The stronger sorting of strategies by sex in the spatial realm is likely due to the fact that biology evolved to deal with this distinction in spatial orienting, whereas we are detecting the impact of this default spatial navigatory preference filtered through an entirely different domain (ie, the EHR). Given this difference, it is remarkable that the effects of this navigatory default are seen so strongly in the physicians in our study. That the physicians in our study still sort robustly by the GI according to sex while navigating the EHR, an artificially constructed environment full of literally thousands of data points that require years of education in order to place in context, speaks to the foundational level of this navigatory difference between females and males.

Despite the significant difference in GI between the sexes, we saw no difference in task performance between females and males overall. No correlation was found between the GI and task performance either overall or at the extremes. This is not a completely unexpected finding. While differences in default navigatory strategies are associated with significant performance differences in spatial navigation tasks, there is more variance in the types and the extent of performance differences exhibited in various types of electronic navigation as in this study [12]. This is likely in part due to the fact that biologic differences would have evolved due to evolutionary pressure from spatial orientation tasks. In addition, there is also the question of the ability to control for confounding variables in the task. Spatial navigation tasks are basic and do not involve higher cognitive reasoning processes; however, the same cannot be said of medical analysis tasks, which integrate cognition and navigatory skills. The task assigned to our subjects involved significant parsing of standardized, simulated ICU patient data, and, as such, diagnostic reasoning is inextricably tied to the eventual task score even though the cases themselves are the same between participants. In line with task load principles, with increasing task difficulty, one by definition will see load (cognitive and/or physical) increase before performance decreases. Therefore, it is plausible that successful completion of the task only partially depended on electronic navigation and may not have been focused or challenging enough to elicit a performance decrease.

### Limitations

One important limitation to this study is that we only measured performance as determined by task completion without assessing the cognitive load and stress incurred during the task. Indeed, this may be the more important metric for future studies given the association of EHR use with burnout and job dissatisfaction. This is especially important given the number of studies that suggest significant differences in metrics potentially related to the EHR between female and male physicians, with female physicians reporting more compassion fatigue, double the rate of burnout, lower job satisfaction, lower pay for comparable work, a larger increase in suicide rate from the baseline population as compared to their male colleagues, and increased time pressure during their work [37-41].

Our study had a variety of other limiting features apart from the inability to measure cognitive load and stress. Given the secondary analysis of the study, we did not capture all other potential sociotechnical confounders such as prior EHR experience from the subjects. As such, our ability to assess whether other variables of a social or environmental nature were also associated with the GI was limited. Perhaps most significantly, the task the physicians performed was not optimized for the detection of eye-tracking variables and introduced a number of unnecessary steps between navigation and scoring given its focus on oral presentations. Physicians spent significant time looking away from their screen to their written notes or the provided signout, which limited the amount of eye tracking performed. Our outcome measure being orally reported data points meant that oral presentation skills, recall, and even handwritten data organization were all introduced as unnecessary confounding variables. While these task limitations may have increased our chance of type II error with regard to detection of a relationship between the GI and task score, we do not expect that they would have increased the chance of type I error with regard to the detected difference in GI between females and males. Finally, while all subjects had some experience with EHRs and specifically were familiar with its use in the context of the activity being simulated, we do not have other demographic information on the subjects such as age, total years of experience with the institution’s specific EHR system, and experience with other forms of electronic data interfaces. Thus, we cannot fully exclude these as additional factors that may serve to confound the observed findings; this highlights the need to further confirm these findings in a dedicated, prospective evaluation with a dedicated assessment of these domains.

### Conclusions

In conclusion, the results of our study presented here, specifically differences in GI, total screen distance traveled, individual saccadic period duration, individual fixation duration, etc, support the conclusion that, as a group, female physicians are more likely to scan longer, more often, and more broadly while male physicians are more likely to fixate on particular areas of interest. Demonstrated differences in GI and other eye-tracking metrics are similar to those seen in other electronic navigatory tasks in which gaze type maps with performance, and also similar to eye movement differences seen in spatial navigatory tasks. This association is concerning as it raises the possibility that physicians of one sex may be disproportionately impacted by design considerations of the EHR interface in a manner that potentially cannot be corrected with end-user training or mitigated over time with increasing familiarity of EHR use.

## Abbreviations

EHR: electronic health record
GI: Gaze Index
ICU: intensive care unit
OR: odds ratio
PGY: postgraduate year
